# Motor Skills as Moderators of Core Symptoms in Autism Spectrum Disorders: Preliminary Data From an Exploratory Analysis With Artificial Neural Networks

**DOI:** 10.3389/fpsyg.2018.02683

**Published:** 2019-01-09

**Authors:** Francesca Fulceri, Enzo Grossi, Annarita Contaldo, Antonio Narzisi, Fabio Apicella, Ilaria Parrini, Raffaella Tancredi, Sara Calderoni, Filippo Muratori

**Affiliations:** ^1^Research Coordination and Support Service, Istituto Superiore di Sanità, Rome, Italy; ^2^Autism Research Unit, Villa Santa Maria Institute, Tavernerio, Italy; ^3^IRCCS Fondazione Stella Maris, Pisa, Italy; ^4^Department of Clinical and Experimental Medicine, University of Pisa, Pisa, Italy

**Keywords:** autism spectrum disorders, motor impairments, motor skills, repetitive behaviors, artificial neural network, Peabody Developmental Motor Scale, preschoolers

## Abstract

Motor disturbances have been widely observed in children with autism spectrum disorder (ASD), and motor problems are currently reported as associated features supporting the diagnosis of ASD in the current Diagnostic and Statistical Manual of Mental Disorders (DSM-5). Studies on this issue reported disturbances in different motor domains, including both gross and fine motor areas as well as coordination, postural control, and standing balance. However, they failed to clearly state whether motor impairments are related to demographical and developmental features of ASD. Both the different methodological approaches assessing motor skills and the heterogeneity in clinical features of participants analyzed have been implicated as contributors to variance in findings. However, the non-linearity of the relationships between variables may account for the inability of the traditional analysis to grasp the core problem suggesting that the “single symptom approach analysis” should be overcome. Artificial neural networks (ANNs) are computational adaptive systems inspired by the functioning processes of the human brain particularly adapted to solving non-linear problems. This study aimed to apply the ANNs to reveal the entire spectrum of the relationship between motor skills and clinical variables. Thirty-two male children with ASD [mean age: 48.5 months (*SD*: 8.8); age range: 30–60 months] were recruited in a tertiary care university hospital. A multidisciplinary comprehensive diagnostic evaluation was associated with a standardized assessment battery for motor skills, the Peabody Developmental Motor Scale-Second Edition. Exploratory analyses were performed through the ANNs. The findings revealed that poor motor skills were a common clinical feature of preschoolers with ASD, relating both to the high level of repetitive behaviors and to the low level of expressive language. Moreover, unobvious trends among motor, cognitive and social skills have been detected. In conclusion, motor abnormalities in preschoolers with ASD were widespread, and the degree of impairment may inform clinicians about the severity of ASD core symptoms. Understanding motor disturbances in children with ASD may be relevant to clarify neurobiological basis and ultimately to guide the development of tailored treatments.

## Introduction

According to the Diagnostic and Statistical Manual of Mental Disorders, fifth edition (DSM-5) (American Psychiatric Association [APA], 2013), autism spectrum disorders (ASD) are a heterogeneous set of neurodevelopmental disorders characterized by deficits in social communication and social interaction along with the presence of restricted and repetitive behaviors. Motor impairments have been widely reported in children with ASD (Fournier et al., 2010), but it is still unclear whether they are represented across the spectrum and whether they are related to the clinical specifiers (i.e., intelligence, language, comorbidity, and associated conditions). The development of reliable motor skills is considered an essential component to enhance communication and social engagement (Iverson, 2010; Soska et al., 2010; Bhat et al., 2011), and motor dysfunctions may influence social development interfering with opportunities for social experiences and social learning (Sacrey et al., 2014). Also, it has been suggested that the impairment in motor skills in children with ASD may also impact their others’ actions understanding (Gallese et al., 2013). Thus, investigating the nature of motor problems in ASD may bring a new perspective into diagnostic and treatment approaches. Indeed, motor impairments have been identified in children with ASD at an early stage of development (Esposito and Venuti, 2008; Phagava et al., 2008; Esposito et al., 2009, 2011; Bhat et al., 2011; Landa et al., 2013; Gima et al., 2018), and a growing research interest has been directed toward investigating the effects of motor intervention on both core and associated symptoms of ASD (Brand et al., 2015; Bremer et al., 2015; Ketcheson et al., 2017; Pan et al., 2017; Bishop and Pangelinan, 2018; Najafabadi et al., 2018).

The motor deficits occur more frequently in children with ASD than in children with typical development (TD) (Fournier et al., 2010; Moseley and Pulvermüller, 2018). Moreover, motor problems are currently reported as associated features supporting the diagnosis of ASD (American Psychiatric Association [APA], 2013). To date, impairments have been highlighted in various motor domains including both gross and fine motor areas, motor coordination, postural control and standing balance (Fournier et al., 2010; Esposito et al., 2011; Longuet et al., 2012; Travers et al., 2013; May et al., 2016). The high variance in findings may suggest a broad spectrum of motor impairments. However, it should be noted that various methodological approaches in different studies could play a role in the heterogeneity of results.

First, different studies have examined motor skills through various instruments (Wilson et al., 2018) including home-video analysis (Gernsbacher et al., 2008; Ozonoff et al., 2008; Phagava et al., 2008; Zappella et al., 2015), parent reports (Kopp et al., 2010; LeBarton and Iverson, 2013; Hedgecock et al., 2018), developmental tests (Landa and Garrett-Mayer, 2006; Landa et al., 2013; Libertus et al., 2014), specific motor batteries as the Movement Assessment Battery for Children (Green et al., 2009; Whyatt and Craig, 2012) and the Peabody Developmental test (Provost et al., 2007; Jasmin et al., 2009; Fulceri et al., 2015), kinematic analysis during walking or prehension movement (Glazebrook et al., 2006; Campione et al., 2016; Eggleston et al., 2017) and electronic balance board (Travers et al., 2013; Stins et al., 2015).

Secondly, differences observed in clinical and demographical features of ASD individuals between studies should be mentioned. In fact, some studies enrolled infants and young children (Landa and Garrett-Mayer, 2006; Provost et al., 2007; Phagava et al., 2008; Esposito et al., 2009; Jasmin et al., 2009), whereas others involved school-aged children, adolescents, and adults (Minshew et al., 2004; Green et al., 2009; Staples and Reid, 2010). Moreover, some studies enrolled children without intellectual disabilities (MacDonald et al., 2013; Miller et al., 2014) whereas others enlisted children with a wide range of cognitive functioning (Vanvuchelen et al., 2007; Green et al., 2009; Fulceri et al., 2015). In this regard, it has been reported that intellectual functioning may be related to motor skills, with low performances associated with reduced motor skills (Mari et al., 2003; Dewey et al., 2007; Dziuk et al., 2007; Vanvuchelen et al., 2007; Dowell et al., 2009; Green et al., 2009; Hilton et al., 2012; Fulceri et al., 2015). However, the largest meta-analysis on this issue to date (Fournier et al., 2010) was unable to unequivocally state the impact of intellectual functioning on motor skills in individuals. Analogously, the relationship between motor impairments and language abilities in children with ASD has not been yet clarified. The observation of motor impairments in children with poor language skills has been related to the coexistence of intellectual impairment (Moseley and Pulvermüller, 2018). However, it should be noted that studies on infants at high familial risk for ASD (infants who have an older sibling with an ASD diagnosis) revealed that the level of fine motor skills in the first years of life was able to predict the language development (LeBarton and Iverson, 2013; Garrido et al., 2017). The interconnection between motor and language skills during development has been clearly defined in children with TD (Libertus and Violi, 2016), and this evidence may be relevant also for children with ASD (Kim, 2008; Bedford et al., 2016; Srinivasan and Bhat, 2016). Finally, the degree of ASD severity has been found to be related to the severity of motor problems (Jasmin et al., 2009; Hilton et al., 2012; MacDonald et al., 2013; Purpura et al., 2016), even if contrasting data have been reported (Zachor et al., 2010; Fulceri et al., 2015).

Thirdly, the traditional statistical analysis approach performed in the studies mentioned above suffered from some criticisms. Indeed, due to the heterogeneity in clinical expression of ASD (i.e., different degree of symptoms severity along with various co-morbidities), it could be possible that traditional “single symptom approach analysis” did not provide comprehensive information. Artificial neural networks (ANNs) are computational adaptive systems inspired by the functioning processes of the human brain particularly adapted to solve non-linear problems (Krogh, 2008; Manning et al., 2014) and to discover unobvious trends and associations among variables. Based on their learning through an adaptive way (i.e., extracting from the available data the information needed to gather a specific task and to generalize the acquired knowledge), the ANNs appear to be a powerful tool for data analysis also in the presence of relatively small samples (Buscema et al., 2015).

In the last years, the ANNs have been increasingly used in medicine (Cocchi et al., 2008; Street et al., 2008; Ayer et al., 2013; Gironi et al., 2013; Peixoto et al., 2015; Toscano et al., 2017), and recently they have also been applied to the ASD field (Buscema et al., 2015; Narzisi et al., 2015; Djemal et al., 2017; Grossi et al., 2017, 2018). Overall, literature findings suggest that the ANNs method may be a strategic approach to grasp the core of the relationship between motor impairments and clinical and developmental features. Therefore, this study aimed to explore the associations between motor skills and clinical/developmental features in a sample of preschoolers with ASD through the ANNs. We hypothesized that the ANNs approach would be able to reveal latent connections among the full spectrum of clinical/developmental variables revealing their simultaneous connections with different domain or degrees of motor impairment.

## Materials and Methods

### Participants

Participants were a selected subset of a group children recruited at ASD unit at the IRCCS Fondazione Stella Maris, a tertiary care university hospital. Each child received a clinical diagnosis of autistic disorder (AD) or pervasive developmental disorder not otherwise specified (PDD-NOS), based on DSM-IV-TR criteria (American Psychiatric Association [APA], 2000) after several days of extensive and comprehensive evaluation. The diagnosis was confirmed by Autism Diagnostic Observation Schedule (ADOS-G; Lord et al., 2000) and all children satisfy criteria for a diagnosis of ASD according to DSM-5 (American Psychiatric Association [APA], 2013). Exclusion criteria were: (a) neurological syndromes or focal neurological signs; (b) significant sensory impairment (e.g., blindness, deafness); (c) anamnesis of severe birth asphyxia, head injury or epilepsy; (d) potential secondary causes of ASD revealed by high-resolution karyotyping, DNA analysis of Fragile-X, or screening tests for inborn errors of metabolism. The demographical and clinical features of the original sample have been previously published (Fulceri et al., 2015). For this study, incomplete data have been excluded, and analyses have been limited to data from children with non-verbal IQ ≥ 70. The final sample consisted of 32 male preschoolers with a diagnosis of ASD and with non-verbal IQ ≥ 70 (age range: 30–60 months) who were evaluated through a standardized assessment battery for the gross and fine motor skills: the Peabody Developmental Motor Scale-Second Edition (PDMS-2; Folio and Fewell, 2000).

**FIGURE 1:**
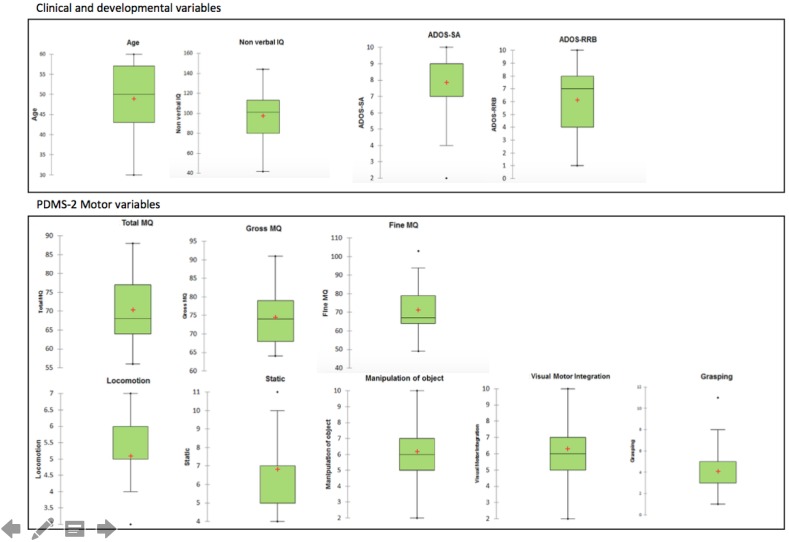
Descriptive statistics (box and whisker plot) of variables on study.

It should be noted that the characteristics of this sample substantially overlapped with those of the sample considered in Fulceri et al. (2015). Descriptive statistics (box and whisker plot) of variables on the study are presented in Figure 1. Table 1 summarized the demographical and clinical features of the sample.

**Table 1:** Demographical and clinical features of participants.

Age in months *mean (SD)*	48.5 (8.8)
Autism Diagnostic Observational Schedule Scores	
ADOS-SA *mean (SD)*	7.9 (2.1)
ADOS-RRB *mean (SD)*	6.1 (2.2)
Module 1	8/32
Module 2	24/32
Non-verbal IQ Scores *mean (SD)*	99.3 (19.8)
Motor intervention	24/32


*ADOS, Autism Diagnostic Observational Schedule; SA, Social Affect domain; RRB, Restricted and Repetitive Behaviors domain.*

This study was carried out following the recommendations of the IRCCS Stella Maris Foundation. The Ethics Committee of IRCCS Stella Maris Foundation approved the study and the head of the ASD unit at the IRCCS Fondazione Stella Maris supervised the project activities. All parents/guardians gave written informed consent in accordance with the Declaration of Helsinki.

### Instruments

#### Motor Assessment

The PDMS-2 is a standardized assessment battery for gross and fine motor skills. This test consists of six subscales: Reflexes, Stationary, Locomotion, Object Manipulation, Grasping, and Visual-Motor Integration. The combination of the results in these subscales provides three quotients: Gross Motor Quotient (Gross MQ), Fine Motor Quotient (Fine MQ), and Total Motor Quotient (Total MQ). Subscales and quotients scores are classified into seven categories: very superior, superior, above average, average, below average, poor, and very poor. At the time of the study, the Italian version of PDMS-2 has not been yet officially edited. Thus, members of the research team who individually translated the source text of the English version of the PDMS-2 and sought agreement between own translations have elaborated an experimental and un-official Italian version of PDMS-2 used in this study. To date, an official Italian version of this test has been provided by Biancotto et al. (2017).

#### Autism Diagnostic Measures

The ADOS-G is a semi-structured assessment to rate the child’s social-communicative impairment and restricted and repetitive behaviors. In this study, only Module 1 (pre-verbal/single words/simple phrases) and Module 2 (flexible phrase speech) were applied. The social affect score (ADOS-SA) and the restricted, repetitive behaviors score (ADOS-RRB) were calculated according to published algorithms (Gotham et al., 2009; Hus Bal and Lord, 2015). According to ADOS scoring, the low scores of ADOS are the expression of less impairment while high scores correspond to a higher impairment.

#### Language Abilities

The level of language abilities of children was evaluated according to the module of ADOS used to assess ASD symptoms. Indeed, the module of ADOS is selected according to the language level of the child. In detail, Module 1 is selected for children who do not use spontaneous phrase speech consistently (defined as non-echoed). Three-word utterances that sometimes involve a verb and that are spontaneous, meaningful word combinations. Module 2 is selected for children with some flexible phrase speech who are not verbally fluent. The operational definition of verbal fluency is the spontaneous, flexible use of sentences with multiple clauses that describe logical connections within a sentence. It requires the ability to talk about objects or events not immediately present (Lord et al., 2000).

#### Cognitive/Developmental Abilities

Several standardized tests (i.e., Leiter International Performance Scale-Revised, Roid and Miller, 1997; Griffiths Mental Development Scale-ER, Luiz et al., 2006; Wechsler Preschool and Primary Scale of Intelligence, Wechsler, 1974) were used to assess intellectual abilities according to clinical features (i.e., age, language level) of children. For this study, only non-verbal IQ scores were considered.

#### Motor Treatment History

Information about psychomotor treatment history were evaluated according to the following “yes/no” parent question: “Has your child ever received a psychomotor intervention?” The psychomotor intervention is an approach for child’s rehabilitation widely used in Italian Care Service for children with ASD. This approach focuses on child’s global development promoting awareness of personal space, sharing a common space during play situations, increasing of attention and development of social interaction. Body and movements are considered principal mediators between child and surrounding. University courses in Italy, train therapists in theoretical framework and techniques of psychomotor intervention.

## Data Analysis

### Linear Correlation Analysis

Pearson correlation analysis was performed between the motor variables (Stationary, Locomotion, Object Manipulation, Grasping and Visual-Motor Integration, Gross MQ, Fine MQ, Total MQ) and the demographical/clinical variables (chronological age, ADOS-SA, ADOS-RRB, ADOS Module 2; ADOS Module 1, Motor Intervention). A *p*-value of <0.05 was considered. Statistical analysis was performed with SPSS package.

Even if recommended in conducting multiple analyses on the same dependent variable, in this study the linear correlation analysis involved multiple comparisons has been performed without considering the Bonferroni correction. Indeed, being an exploratory analysis looking for important associations among many independent variables, there is no oblige to apply this type of adjustment. While the number of correlations among *N* independent variables and a dependent variable is equal to *N* - 1, in case of absence of a dependent variable the number of correlations among all independent variables tends to increase exponentially with their number (*N*^∗^*N* - 1/2) penalizing inevitably the significance of correlation indexes even when they are substantially high.

To limit the false discovery rate, the procedure described in Benjamini and Yekutieli (2001) has been applied obtaining that all *r*-values above 0.34 reported in the Table 2 remain significant.

**Table 2:** Linear correlation analysis.

PDMS-2	Age	Autism Diagnostic Observational Schedule				Non-verbal IQ	Motor Intervention
				
		Social affect	Repetitive behaviors	Module 1	Module 2		
**Quotients**							
Total	-0.175	-0.086	**-0.394**^∗^	-0.054	0.054	**0.575**^∗∗^	-0.027
Gross	-0.291	-0.076	-0.309	-0.084	0.084	**0.384**^∗^	-0.097
Fine	-0.011	-0.067	**-0.358**^∗^	-0.010	0.010	**0.574**^∗∗^	0.040
**Subscales**							
Stationary	**-0.395^∗^**	0.050	-0.199	-0.105	0.105	0.289	0.115
Locomotion	-0.275	-0.044	-0.285	-0.288	0.288	0.181	-0.153
Object Manipulation	0.067	-0.192	-0.201	0.121	-0.121	0.322	-0.253
Grasping	-0.026	0.016	-0.297	-0.024	0.024	**0.392**^∗^	0.037
Visual-Motor Integration	0.008	-0.142	-0.329	0.009	-0.009	**0.622**^∗∗^	0.033


*PDMS-2, Peabody Developmental Motor Scale; IQ, non-verbal IQ; Module 1, non-verbal module of ADOS; Module 2, verbal module of ADOS; Intervention, history of motor intervention; ^∗^*p* < 0.05; ^∗∗^*p* < 0.01. Significant data are in bold.*

### Artificial Neural Networks Analysis

#### General Description

The detailed description of Auto-CM was provided in the Supplementary Material. The Auto Contractive Map (Auto-CM) system is a fourth generation unsupervised ANNs which has already been demonstrated to outperform several other unsupervised algorithms in a heterogeneous class of tasks (Buscema and Sacco, 2017).

Through a specific learning algorithm, the Auto-CM system detects a square matrix of “similarities” (named weights) among the variables considered. Once the Auto-CM weights matrix is obtained, it is then filtered by a minimum spanning tree algorithm (Kruskal, 1956; Fredman and Willard, 1990). The minimum spanning tree shows among the full spectrum of possible ways to connect the variables in a tree, the shortest combination. Based on the minimum spanning tree theory, the Auto-CM reveals the connections among variables providing a graph in which the distances among variables reflect their bonding strength (weights) (Buscema and Grossi, 2008; Buscema et al., 2008). A detailed description of the minimum spanning tree concept is provided in the Supplementary Material.

This data-mining model aims to detect hidden trends and unobvious associations among variables. Indeed, this algorithm provides a semantic connectivity map in which non-linear associations are preserved, and explicit connection schemes are described. This approach provides the map of relevant connections between and among variables and the principal hubs of the system. Hubs can be defined as variables with the maximum amount of connections in the map. The Auto-CM does not pose the initial weights randomly. Conversely, the Auto-CM starts with the same value. Thus, the resulting graph is reproducible along many runs. In other words, the Auto-CM ‘spatializes’ the correlation among the variables (‘closeness’) and the graph identifies only the relevant associations organizing them into a coherent picture. The “central node” is the inner node that remains after bottom-up recursively pruning away the “leaves” nodes.

#### Artificial Neural Networks Applied to Our Dataset

The 12 continuous variables (Total MQ, Gross MQ, Fine MQ, Stationary, Locomotion, Object Manipulation, Grasping, Visual-Motor Integration, Non-verbal IQ, ADOS-SA, ADOS-RRB, Age) and the two nominals (ADOS Module, Motor Intervention) were included.

In pre-processing, each continuous variable was transformed in two variables either named “high” and “low” based on the median score of each variable. This pre-processing was necessary to evidence the connections of each variable when their values were high or low (that is above or below the median score of each variable). Consequently, the projection of the original variable occupied two positions in the space: one related to high values and the other related to low values. In the map, these two conditions were named “high” and “low.” It should be noted that in non-linear systems, the position of a variable according to its high and low values is not necessarily symmetric in the map.

For the nominal variables, we merely provided the following three variables: Module 1, Module 2, and Motor Intervention.

### Benchmarking Analysis

The Auto-CM and the self-organizing maps are systems for unsupervised data mining. However, it should be noted that the self-organizing maps are mainly directing the clustering individuals rather than variables. To handle a benchmarking analysis the principal component analysis (PCA) and hierarchical agglomerative clustering (HAC) were carried out with XLSTAT package 2018. In detail, PCA is mathematically defined as an orthogonal linear transformation that transforms the data to a new coordinate system such that the greatest variance by any projection of the data comes to lie on the first coordinate (called the first principal component), the second greatest variance on the second coordinate, and so on. PCA is theoretically the optimum transform for given data in least square terms. AHC is one of the most popular clustering methods which seeks to build a hierarchy of clusters with a “bottom-up” approach: each observation starts in its own cluster, and pairs of clusters are merged as one moves up the hierarchy. This method works from the dissimilarities between the objects to be grouped together producing the so-called “dendrogram,” which shows the progressive grouping of the data. It is then possible to gain an idea of a suitable number of classes into which the data can be grouped.

Results of this benchmarking analysis allowed us to compare findings from the Auto-CM approach with findings from the traditional statistical approach.

## Results

### Motor Skills According to the PDMS-2 (Figure 2)

Total QM, Gross QM, Fine QM mean scores were into the “Poor class”; any child had individual Total QM score in the Average class; one child and two children had, respectively, Gross QM and Fine QM in the Average class. Almost all subscales mean scores were into the “Below Average class” or the “Poor class.”

**FIGURE 2:**
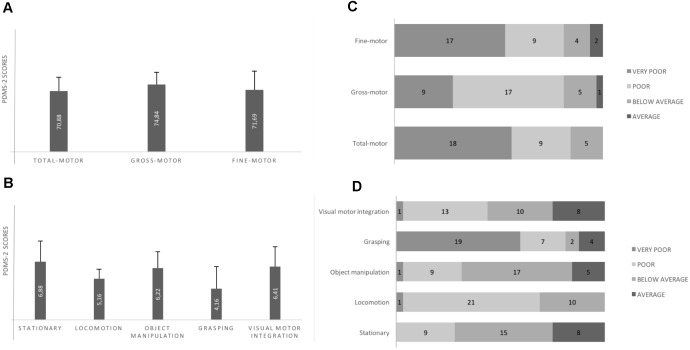
Motor performance according to PDMS-2. **(A)** All quotient mean scores are into the Poor class (standard scores for the Poor class range from 70 to 79). **(B)** Locomotion and Grasping subscales scores are into the Poor class (standard scores for the Poor class range from 4 to 5); Static, Object Manipulation and Visuo-Motor Integration are into the Below Average class (standard scores for Below Average class range from 6 to 7). **(C)** Individual motor quotients: any child shows total motor quotient in the Average class (standard scores for Average class range from 8 to 12. **(D)** Individual motor subscales: Grasping and Locomotion subscales (i.e., the more impaired subscales according to the mean scores of the sample) appear to be differently impaired. Any child shows Locomotion abilities into the Average class; many children show Grasping abilities into the Very Poor class (standard scores for the Very Poor class range from 1 to 3). Error bars are standard deviation.

### Linear Correlation Analysis

Motor quotients (Total QM and Fine QM) were negatively correlated with “ADOS-RRB.” Motor quotients (Total QM and Fine QM) and motor subscales (Grasping and Visual-Motor Integration) were positively correlated with “Non-verbal IQ.” Stationary subscores were negatively correlated with chronological age. Results are summarized in Table 2.

### Artificial Neural Networks Analysis (Figure 3)

**FIGURE 3:**
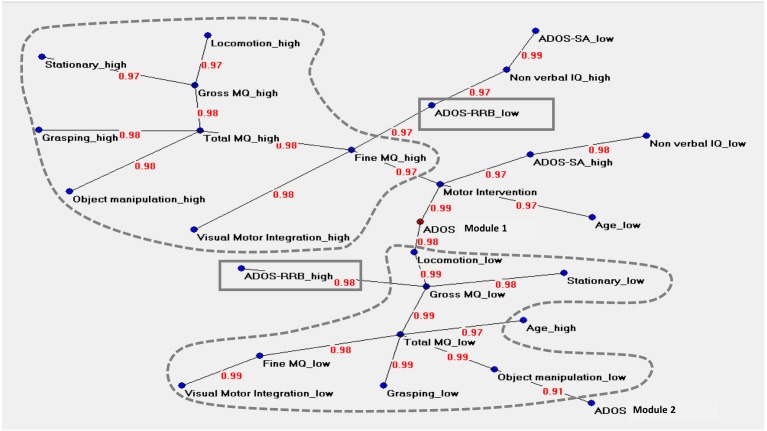
Auto neural networks map. The figure shows the relevant connections among the variables. Each variable is described by two different forms named “high” and “low,” i.e., above or below the median score of each variable. The variable ‘ADOS Module 1’ is the central node of the network. The motor variables indicating a very low level of motor functioning (named “low”) or a less impaired level of motor functioning (named “high”) are strongly connected between them; the two clusters are signed with dotted lines. The upper area is characterized by high values in motor functioning, where ‘Total MQ high’ and ‘Fine MQ high’ act as hubs. ‘Total MQ high’ coordinates a cluster of motor high variables (locomotor, stationary, grasping, object manipulation) while ‘Fine MQ’ is related to ‘Visual-Motor Integration high’ on one side and with ‘ADOS RRB low’ on the other side. This cluster is located close to the variables indicating a high level of cognitive skills and high social functioning. The lower area shows an analog structure of interconnections in opposite sense. ‘Total MQ low’ is the principal hub; ‘ADOS RRB high’ is directly connected with ‘Gross MQ low.’ ‘Total MQ low’ related to the high chronological age. The clinical variables ‘ADOS-SA’ (“high” and “low”) and ‘Non-verbal IQ’ (“high” and “low”) are not directly connected with any motor variables. The variable ‘Age low’ is directly connected with the variable ‘Motor Intervention.’ The variable ‘Motor Intervention’ is located at approximately the same distance between the two-motor clusters. The link strength’s values are presented in red.

The graph in Figure 3 described the central node of the network (ADOS Module 1) and two main areas. The upper area was characterized by values indicating a less impaired level of motor functioning (named “high”), where Total MQ high and Fine MQ high variables acted as hubs. Total MQ high coordinated a cluster of motor variables (locomotor high, stationary high, grasping high, object manipulation high) while Fine MQ high was related to following variables: visual-motor integration high, ADOS RRB low, and motor intervention.

The lower area of the graph showed an analog structure of interconnections in the opposite sense. The motor variables indicating a more impaired level of motor functioning (named “low”) were strongly connected between them. Total MQ low was the principal hub of this cluster of variables. This cluster was also directly related with the following clinical variables: ADOS Module 1, Age high and ADOS Module 2. The ADOS RRB high variable was directly connected with Gross MQ low variable. The direct associations between motor variables and ADOS RRB variable in both the two areas indicated a non-casual association between ASD severity and motor function.

The clinical variables ADOS-SA (“high” and “low”) and Non-verbal IQ (“high” and “low”) were not directly connected with any motor variables. The “Age low” was directly connected to the variable “Motor Intervention.”

### Benchmarking Analysis (Figures 4, 5)

The PCA provided a map in which the two clusters containing ADOS RRB “high” and “low” present a perfect symmetry. Analogously, the AHC provided a dendrogram in the two clusters containing ADOS RRB high and low show a perfect symmetry.

**FIGURE 4:**
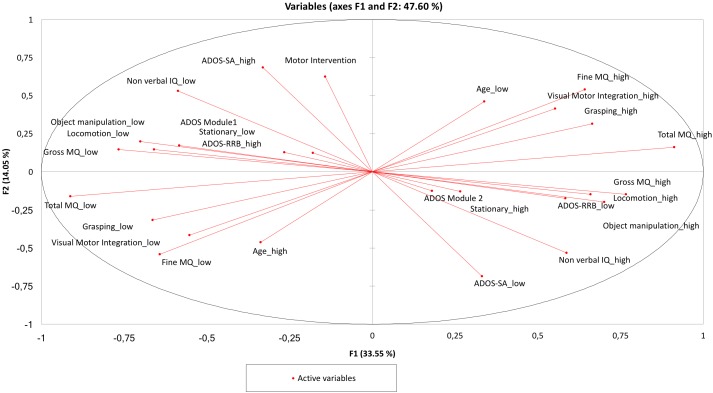
Principal component analysis (PCA). The two clusters containing ADOS RRB “high” and “low” are in a perfect symmetry. The cluster around ADOS RRB “high” indicates the following hierarchy: “ADOS Module 1,” “Stationary low,” “Object Manipulation low,” “Locomotion low,” “Gross MQ low,” and “Non-verbal IQ low,” and in the same way the cluster around ADOS RRB “low” shows the following hierarchy: “ADOS Module 2” “Stationary high,” “Object Manipulation high,” “Locomotion high,” “Gross MQ high,” and “Non-verbal IQ high.”

**FIGURE 5:**
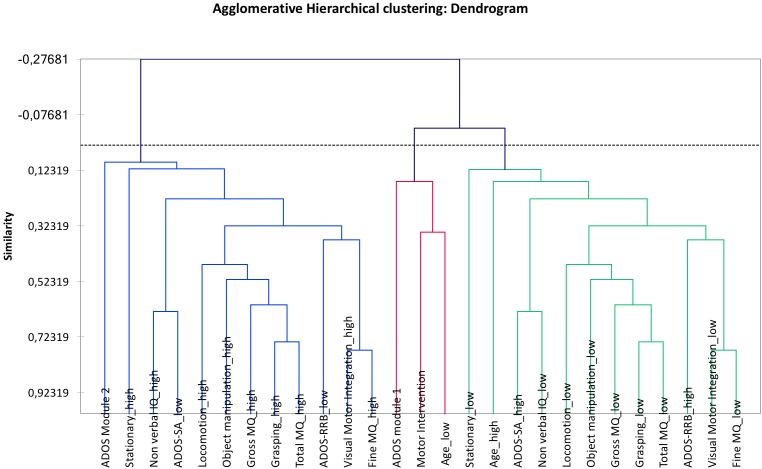
Agglomerative hierarchical clustering (AHC). The two clusters containing ADOS RRB “high” and “low” are in a perfect symmetry. The two clusters around ADOS RRB are: “visual-motor integration low” and “ADOS RRB high”; “visual-motor integration high” and “ADOS RRB low.”

Compared with PCA and HAC analysis, the Auto-CM analysis highlighted the asymmetry of the two clusters departing from ADOS RRB high and ADOS RRB low as one could expect from non-linear mathematics. The closest variable to ADOS RRB low is fine MQ high, while the closest variables to ADOS RRB high is gross MQ low. Asymmetry apart, Auto CM shows the relevance of motor component for other variables. This feature is missed with PCA and ACH.

## Discussion

This study aimed to explore the associations between motor skills and clinical/developmental features in a sample of preschoolers with ASD through the application of ANNs analysis. We hypothesized that the ANNs could reveal simultaneous connections among the full spectrum of clinical/developmental variables.

First, the ANNs revealed that the Module 1 of the ADOS (designed for children whose spontaneous language is primarily in single words or preverbal) was the central node of the network: thus, the lower level of expressive language development was the most shared clinical feature among participants. This finding did not seem surprising since the Module 2 of the ADOS have been administrated to 25% of participants (see Table 2). However, the AANs revealed the direct connection between the lowest level of language skills of participants (i.e., Module 1) and the cluster of motor variables indicating the lowest motor functioning (see Figure 3). This finding acquired a greater relevance considering the growing literature suggesting a relationship between motor and language skills in children with ASD (Bhat et al., 2011; LeBarton and Iverson, 2013; Libertus and Violi, 2016). Motor impairments also emerged in children with some flexible phrase speech (i.e., Module 2 of the ADOS) suggesting that the relationship between motor and language skills may be not linear. It should be noted that our findings were limited by the lack of a direct and structured assessment of children’s language functioning. Thus, future studies aimed at investigating the interconnection between language and motor skills in individuals with ASD should provide a more detailed measure of language abilities, and they should also include children with several different degrees of language functioning.

Second, consistent with the results of the linear correlation analysis, the ANNs detected the association between the motor functioning and the repetitive behaviors in children with ASD. More specifically, the highest impaired motor skills were associated with the highest rate of RRB (see Figure 3). Our findings confirmed previous studies (Radonovich et al., 2013; Ravizza et al., 2013; Purpura et al., 2016; Uljarević et al., 2017) supporting the connection between motor and repetitive behaviors domains. Moreover, our results were consistent with some findings from neuroimaging studies according to which RRB and motor skills share common neural underpinnings as the basal ganglia, the cerebellum and the associated cortico-subcortical circuitries, including the striatum and thalamus (Lopez et al., 2005; Lam and Aman, 2007; Leekam et al., 2011).

Third, the AANs provided new insights into the simultaneous connections among motor skills and the full spectrum of variables including cognitive and social abilities. Consistent with previous data (Mari et al., 2003; Dewey et al., 2007; Green et al., 2009; Fulceri et al., 2015), linear correlation analysis revealed that the increase of motor skills, mainly fine motor skills, was associated with the increase of cognitive abilities. The ANNs approach did not disconfirm this finding but highlighted two different motor clusters differently connected with clinical and developmental variables (see Figure 3). Indeed, the motor variables indicating the lowest impaired motor skills were located close to the variables indicating a high level of cognitive skills and high social functioning. The variables indicating the highest impaired motor skills were located close (although to a lesser degree than the previous ones) to the variables indicating a low level of cognitive skills and low social functioning. According to this picture, children with the best motor functioning should be expected to present the lowest impairment in cognitive functioning and ASD severity. This latter picture seemed to emphasize the broad body of literature on this topic (Mari et al., 2003; Dewey et al., 2007; Green et al., 2009; Hilton et al., 2012; Fulceri et al., 2015) suggesting that motor skills may be accordingly influenced by different degree of impairment in clinical and developmental abilities.

There is a lack of strong closeness between the motor clusters and the variables indicating a different degree of social impairment. This finding led us to suppose that the relevance of motor development in the research field of ASD may be prevalent for RRB domain rather than in SA domain occurring regardless of the intellectual functioning. However, any interpretation of these preliminary data should be made with great caution given the methodology of the current work that may present limitations.

Fourth, the ANNs highlighted and expanded the relationship between motor skills and chronological age revealed from linear correlation analysis. Indeed, even if the ANNs approach did not detect a direct relationship between motor skills and chronological age, it should be noted that the cluster of highest impaired motor skills was located close to the variable “high chronological age,” supporting the hypothesis that motor abilities may worsen over time (Minshew et al., 2004; Lloyd et al., 2013). However, it is essential to consider that the cross-sectional nature of the current study implies caution in the interpretation of age-related phenomena, as they do not derive from intra-individual measures of change. Therefore, further longitudinal studies should be performed to strengthen these results.

Finally, the variable “motor intervention” was located at approximately the same distance between the two-motor clusters, and it was directly related to the lowest chronological age and the highest social affect impairment. This finding could be explained in the light of the Italian public care services context. Indeed, psychomotor intervention is among the early rehabilitative treatments offered to young children with a diagnosis of ASD, often regardless of their motor functioning. The lack of proximity between the variable “motor intervention” and “highest chronological age” (i.e., older children) was quite unexpected. Indeed, since this treatment is offered at an early age of development, the older children should also have practiced it. Our methodology limited further interpretations. Indeed, the question relating to psychomotor treatment was extremely vague and the answers of parents, including possible memory bias, were not investigated in more detail.

Several limitations should be discussed. The most relevant is undoubtedly the constrained data-set that strongly limits wide-ranging conclusions. The sample of participants was limited to a group of 32 male children with non-verbal IQ ≥ 70. It should be noted that in this study, only non-verbal IQ scores ≥ 70 were considered and that several standardized tests were used to assess intellectual abilities according to clinical features. Even if a numbers of exclusion criteria have been defined, it should be noted that it could be problematic to artificially define cutoff point of 70 of non-verbal IQ for ruling out co-morbidity of intellectual disability based on the age range (30–60 months) and the issues of testing non-verbal/verbal IQs for children (Bishop et al., 2015). However, the adaptive learning algorithms of inference, based on the principle of a functional estimation like ANN, has been reported as a strategical approach to overcome the problem of dimensionality (Buscema et al., 2015). Different from the classical statistics, the ANNs can manage complexity even with relatively small samples and to the subsequent unbalanced ratio between variables and records. Among other limitations, it should be noted that the PDMS-2 motor scores could reflect not only the motor impairment but also the child’s difficulties to attend the standardized context. However, the PDMS-2 has been widely and successfully used in children with ASD (Provost et al., 2007; Vanvuchelen et al., 2007; Jasmin et al., 2009; Zachor et al., 2010), due to the lack of time trials and the less cognitive demand compared to other motor assessment tools (Vanvuchelen et al., 2007).

Another substantial limitation of our findings relates to the lack of a typically developing control group. Indeed, even if the PDMS-2 is a standardized clinical tool providing a judgment of motor skills compared to a control sample, the lack of a typically developing group is a methodological criticism that should be underscored. Moreover, also the lack of a control group consisting of preschoolers with other neurodevelopmental disorders (e.g., intellectual disability, attention deficit hyperactivity disorder) should be mentioned since it prevents us to consider the motor abnormalities we detected as specific of ASD. Thus, further research needs to be performed to confirm our findings and to determine the value of motor impairments in ASD compared to those of children with other neurodevelopmental disorders. Also, this study is limited to male children with ASD. In the literature, the issue of gender differences in ASD has been widely discussed. However, even if it has been hypothesized an impaired motor functioning in females with ASD (Kopp et al., 2010), the results of this study are not able to contribute to this discussion.

## Conclusion

In conclusion, this study revealed the pervasiveness of motor impairments in ASD and implemented a machine learning approach to highlight how it is related to repetitive behaviors and low level of expressive language as well as cognitive and social skills. Considering the above-mentioned limitations, this study provided a detailed delineation of the Auto-CM model for unsupervised ANN and its implementation for data mining to discover the “hidden” relationships among multidimensional variables with clusters on a map. This represents a novel approach to understanding the complexity of a key “impairment” measure in ASD such as motor skills and how it is related to other critical measures that define ASD. Findings suggests that motor skills may be a moderator of clinical or developmental features in young children with ASD and that the systematic observation of motor development in ASD may improve the knowledge about clinical and neurobiological involvement as well as guide development of tailored treatments.

## Author Contributions

FF, EG, AC, SC, and FM contributed to the study conception and design, analysis and interpretation of data, to the writing of the manuscript, and to its critical revision. FF, AN, FA, RT, and IP contributed to the acquisition of data, and to the writing of the manuscript and its critical revision. All authors have approved the final version of the manuscript and are accountable for the work described.

## Conflict of Interest Statement

The authors declare that the research was conducted in the absence of any commercial or financial relationships that could be construed as a potential conflict of interest.
