# Supplementary material for: Motor Skills as Moderators of Core Symptoms in Autism Spectrum Disorders: Preliminary Data From an Exploratory Analysis With Artificial Neural Networks
Source: Front Psychol. 2019 Jan 9;9:2683. doi: 10.3389/fpsyg.2018.02683 (PMC6333655; doi:10.3389/fpsyg.2018.02683)
Supplement: Supplementary file 1 [file Table_1.DOCX]

Supplementary Material

Motor skills as moderators of core symptoms in autism spectrum disorder: insights from the Artificial Networks approach

Francesca Fulceri, Enzo Grossi, Annarita Contaldo, Antonio Narzisi, Fabio Apicella, Ilaria Parrini, Raffaella Tancredi, Sara Calderoni^*^, Filippo Muratori

*** Correspondence:** [scalderoni@fsm.unipi.it](mailto:scalderoni@fsm.unipi.it)

**Auto Contractive Map (Auto-CM)**

The Auto-Contractive Map (Auto-CM) is a new paradigm of Artificial Neural Networks (ANNs). The Auto-CM differs from the traditional ANNs in a number of important aspects, mainly on the initialization of the weights and the conditions under which convergence is achieved.

**The mathematical representation of the AutoCM**

Before we build the contractive map (CM), all the connections can be initialized either by equal values or by randomly selected values. The best practice is to initialize all the connections with the same positive value, close to zero.

The learning algorithm of CM may be summarized in four steps:

1. Signal Transfer from the Input into the Hidden layer;
2. Adaptation of the connections value between the Input layer and the Hidden layer; *
3. Signal Transfer from the Hidden layer into the Output layer; *
4. Adaptation of the connections value between the Hidden layer and the Output layer.

(*): step 2 and 3 may take place in parallel.

We define as *m*[*^s^*] the units of the Input layer (sensors), scaled between 0 and 1, as *m*[*^h^*] the units of the Hidden layer and as *m*[*^t^* ] the units of the Output layer (system target). We define **v** the vector of monodedicated connections, **w** the matrix of the connections between Hidden layer and Output layer, and *n* the discrete time of the weights evolution.

The signal forward transfer equations and learning are:

**a.** Signal transfer from the Input to the Hidden:

(1) $m_{i_{(n)}}^{[h]}=m_{i_{(n)}}^{[s]}\left(1-\frac{v_{i_{(n)}}}{C}\right)$ where *C* = Positive real number, named Contractive Factor.

**b.** Adaptation of the connections through the $\Delta v_{i_{(n)}}$trapping the energy difference generated by the equation (1):

(2) $\Delta v_{i_{(n)}}=\left(m_{i_{(n)}}^{[s]}-m_{i_{(n)}}^{[h]}\right)\cdot\left(1-\frac{v_{i_{(n)}}}{C}\right)$;

(3) $v_{i_{(n+1)}}=v_{i_{(n)}}+\Delta v_{i_{(n)}}$;

**c.** Signal transfer from the Hidden to the Output:

(4) $Net_{i_{(n)}}=\sum_{j}^{N} m_{j_{(n)}}^{[h]}\cdot\left(1-\frac{w_{i,j_{(n)}}}{C}\right);$

(5) $m_{i_{(n)}}^{[t]}=m_{i_{(n)}}^{[h]}\left(1-\frac{Net_{i_{(n)}}}{C}\right)$.

**d.** Adaptation of the connections $w_{i,j_{(n)}}$through the $\Delta w_{i,j_{(n)}}$ trapping the energy differences generated by the equation (5):

(6) $\Delta w_{i,j_{(n)}}=\left(m_{i_{(n)}}^{[h]}-m_{i_{(n)}}^{[t]}\right)\cdot\left(1-\frac{w_{i,j_{(n)}}}{C}\right)\cdot m_{j_{(n)}}^{[h]}$;

(7) $w_{i,j_{(n+1)}}=w_{i,j_{(n)}}+\Delta w_{i,j_{(n)}}$

The value $m_{j_{(n)}}^{[h]}$ of (6) is used to proportion the change of the connection $w_{i,j_{(n)}}$ to the energy liberated by the node $m_{j_{(n)}}^{[h]}$ in favour of node $m_{i_{(n)}}^{[t]}$.

The learning process, considered as the adjustment of the connections in relation to the minimization of Energy, corresponds to the continuous acceleration and deceleration of velocities of the signals inside the ANN connection matrix.

This can be mathematically described with the CM convergence equation:

(8) $\lim_{n\to\infty} v_{i_{(n)}}=C$.

when $v_{i_{(n)}}=C$, then $\Delta v_{i_{(n)}}=0\text{ (eq 2)}$, and $m_{i_{(n)}}^{[h]}=0\text{ (eq 1)}$ and, consequently, $\text{ }\Delta w_{i,j_{(n)}}=0\text{ (eq 6)}$.

We define four new variables that play a key role during the AutoCM learning process:

1. $\varepsilon$ is the contractive factor of the first layer of AutoCM weights:

$$\varepsilon=1-\frac{v_{i_{(n)}}}{C}.$$

2. $\eta$is the contractive factor of the second layer of AutoCM weights:

$$\eta=1-\frac{w_{i,j_{(n)}}}{C}$$

3. $\phi$is the contractive factor between the Hidden nodes and the Input nodes:

$$\phi=m_{i_{(n)}}^{[s]}-m_{i_{(n)}}^{[h]};$$

4. $\lambda$ is the contractive factor between the Output nodes and the Hidden nodes:

$\lambda=m_{i_{(n)}}^{[h]}-m_{i_{(n)}}^{[t]}$.

Then we demonstrate how $\Delta v_{i_{(n)}}$ changes during the CM learning phase.

Let us suppose that:

$\frac{v_{i_{(n)}}}{C}=1-\varepsilon,\text{ where }\varepsilon\text{ is a small positive real number close to zero.}$;

Equation (2) can be rewritten as:

(2a) $\Delta v_{i_{(n)}}=(m_{i_{(n)}}^{[s]}-m_{i_{(n)}}^{[s]}\cdot(1-\frac{v_{i_{(n)}}}{C}))\cdot(1-\frac{v_{i_{(n)}}}{C})=m_{i_{(n)}}^{[s]}\cdot\frac{v_{i_{(n)}}}{C}\cdot(1-\frac{v_{i_{(n)}}}{C});$

Since $\frac{v_{i_{(n)}}}{C}=1-\varepsilon$, then:

(2b) $\Delta v_{i_{(n)}}=m_{i_{(n)}}^{[s]}\cdot(1-\varepsilon)\cdot(1-(1-\varepsilon))=m_{i_{(n)}}^{[s]}\cdot(1-\varepsilon)\cdot\varepsilon.$

Equation (2b) shows the parabolic dynamics of $\Delta v_{i}$.

Considering (2b) we can write:

(2c) $\Delta v_{i_{(n)}}<\varepsilon$.

Equation (2c) means that the increment of $\Delta v_{i_{(n)}}$will always be smaller than the quantity that $v_{i_{(n)}}$needs to reach up to *C.*

At this point we can re-write the equation (3) as:

(3a) $v_{i_{(n+1)}}=C\cdot(1-\varepsilon)_{i_{(n)}}+m_{i_{(n)}}^{[s]}\cdot(1-\varepsilon)\cdot\varepsilon.$

Consequently:

(3b) $\lim_{\varepsilon\to0} v_{i_{(n)}}=C$

Further, the contractive factor of the equations (1) and (5) suggests that:

(1-5) $m_{i_{(n)}}^{[t]}<m_{i_{(n)}}^{[h]}<m_{i_{(n)}}^{[s]}$;

In fact:

(1a) $m_{i_{(n)}}^{[h]}=m_{i_{(n)}}^{[s]}\cdot\varepsilon;$

and:

(5a) $m_{i_{(n)}}^{[t]}=m_{i_{(n)}}^{[h]}\cdot\varepsilon\cdot(1-Net_{i_{(n)}}).$

Now it is possible describe the relationship between$\Delta v_{i_{(n)}}\text{ and }\Delta w_{i,j_{(n)}}$.

From the equation (1-5) we can suppose that:

(1b) $m_{i_{(n)}}^{[h]}=m_{i_{(n)}}^{[s]}-\phi;\text{ where }\phi\text{ is a small positive real number close to 0}$ ;

And

(5b) $m_{i_{(n)}}^{[t]}=m_{i_{(n)}}^{[h]}-\lambda;\text{ where }\lambda\text{ is a small positive real number close to 0}$ ;

(5c) $m_{i_{(n)}}^{[t]}=m_{i_{(n)}}^{[t]}-(\phi+\lambda).$

At this point equation (2) can be rewritten as:

(2d) $\Delta v_{i_{(n)}}=(m_{i_{(n)}}^{[s]}-(m_{i_{(n)}}^{[s]}-\phi))\cdot(1-\frac{v_{i_{(n)}}}{C})=\phi\cdot(1-\frac{v_{i_{(n)}}}{C})=\phi\cdot\varepsilon.$

In a similar way we can rewrite the equation (6):

(6a) $\Delta w_{i,j_{(n)}}=((m_{i_{(n)}}^{[s]}-\phi)-(m_{i_{(n)}}^{[s]}-(\phi+\lambda))\cdot(m_{i_{(n)}}^{[s]}-\phi)\cdot(1-w_{i,j_{(n)}})=\lambda\cdot(m_{i_{(n)}}^{[s]}-\phi)\cdot(1-w_{i,j_{(n)}}).$

Now we can rewrite $\frac{w_{i,j_{(n)}}}{C}=1-\eta$ (where $\eta$ has to be a positive real number smaller than 1).

So :

(2e) $\Delta v_{i_{(n)}}=\phi\cdot\varepsilon;$

And

(6b) $\Delta w_{i,j_{(n)}}=\lambda\cdot(m_{i_{(n)}}^{[s]}-\phi)\cdot\eta;$

Considering the equation (5a) in this form:

(5b) $\begin{matrix} m_{i_{(n)}}^{[s]}\cdot\varepsilon\cdot Net_{i_{(n)}}=m_{i_{(n)}}^{[s]}\cdot\varepsilon-m_{i_{(n)}}^{[t]}; \\ Net_{i_{(n)}}=\frac{m_{i_{(n)}}^{[s]}\cdot\varepsilon-m_{i_{(n)}}^{[t]}}{m_{i_{(n)}}^{[s]}\cdot\varepsilon}=\frac{m_{i_{(n)}}^{[s]}\cdot\varepsilon-(m_{i_{(n)}}^{[s]}\cdot\varepsilon-\lambda)}{m_{i_{(n)}}^{[s]}\cdot\varepsilon}=\frac{\lambda}{m_{i_{(n)}}^{[s]}\cdot\varepsilon}. \end{matrix}$

It is now possible to estimate the $\lambda$ contractive factor between Hidden and Output units:

(5c) $\lambda=m_{i_{(n)}}^{[s]}\cdot\varepsilon\cdot Net_{i_{(n)}}$.

From (5c) we can write:

(5d) $\lambda=m_{i_{(n)}}^{[s]}\cdot(1-\frac{v_{i_{(n)}}}{C})\cdot Net_{i_{(n)}}$

and so:

(5e) $\mathop{\lim\lambda=0}\limits_{v_{i_{(n)}}\to C}$.

We can substitute (5c) in (6b):

(6c) $\Delta w_{i,j_{(n)}}=m_{i_{(n)}}^{[s]}\cdot\varepsilon\cdot Net_{i_{(n)}}\cdot(m_{i_{(n)}}^{[s]}-\phi)\cdot\eta;$

Since $m_{i_{(n)}}^{[s]}-\phi=m_{i_{(n)}}^{[s]}\cdot\varepsilon$, then

(6d) $\Delta w_{i,j_{(n)}}=(m_{i_{(n)}}^{[s]}\cdot\varepsilon)^{2}\cdot Net_{i_{(n)}}\cdot\eta;$

And

(6e) $\mathop{\lim\Delta w_{i,j_{(n)}}=0}\limits_{\varepsilon\to0}$

Considering equation (7):

(7a) $w_{i,j_{(n+1)}}=C\cdot(1-\eta)_{i,j_{(n)}}+(m_{i_{(n)}}^{[s]}\cdot\varepsilon)^{2}\cdot Net_{i_{(n)}}\cdot\eta;$

From (7a) we can conclude:

(7b) $\mathop{\lim w_{i,j_{(n)}}=C-C\cdot\eta_{i,j_{(n)}}}\limits_{\varepsilon\to0}$

So this means that at the beginning of training the Input and Hidden units will be very similar (equation (1)), and, consequently, $\Delta v_{i_{(n)}}$will be very small (equation (2e)), while for the same reason$\lambda$, initially will be large (equation (5c)) and $\Delta w_{i,j_{(n)}}$larger than $\Delta v_{i_{(n)}}$(equation (5c)).

During the training, while $v_{i_{(n)}}$slowly increases, $m_{i_{(n)}}^{[h]}$ decreases, so $\phi$increases and, consequently, $\Delta w_{i,j_{(n)}}$ continues to decrease monotonically ($\lambda$ becomes smaller, see equation (5c)) and $\Delta v_{i_{(n)}}$increases faster. When $\lambda$ becomes close to zero, $m_{i_{(n)}}^{[h]}$is only slightly larger that $m_{i_{(n)}}^{[t]}$(see equation (5b)). At this point, $\Delta v_{i_{(n)}}$is on the global maximum of the equation$(1-\varepsilon)\cdot\varepsilon$ (see (2b)) and after this critical point $\Delta v_{i_{(n)}}$will decrease symmetrically toward zero.

**Auto CM: Theoretical consideration**

Auto Contractive Maps do not behave as regular artificial neural networks (ANN):

1. Learning starts from all connections initialised with the same value. So they do not suffer the problem of the symmetric connections.
2. During training, they develop connections for only positive values. Therefore, Auto CM does not have inhibitory relations among nodes, but only different strengths of excitatory connections.
3. Auto CM can learn in hard conditions, that is, when the connections of the main diagonal of the second connection matrix are removed. When the learning process is organized in this way, Auto CM seems to find specific relationships between each variable and any other. Consequently, from an experimental point of view, it seems that the ranking of its connections matrix is equal to the ranking of the joint probability between each variable and the others.
4. After learning, any input vector, belonging to the training set, will generate a null output vector. So, the energy minimization of the training vectors is represented by a function through which the trained connections absorb the input training vectors completely. Auto CM seems to learn to transform itself in a dark body.
5. At the end of the training phase (), the components of the weights vector **v** reach the same value:

(8) $\lim_{n\to\infty} v_{i_{(n)}}=C$ **.**

The matrix **w**, then, represents the CM knowledge for the entire dataset.

It is possible to transform the **w** matrix in a probabilistic joint association between the variables *m***:**

(9)

(10)

The new matrix **p** can be considered as the probability of transition from any state-variable to any other:

(11) .

g. At the same time, the matrix **w** can be transformed into a non-Euclidean distance metric (u-metric), with the main diagonal of the **w** matrix fixed at value N.

If we consider N as a limit value for all the weights of the **w** matrix, we can write:

(12)

The new matrix **d** is also a squared symmetric matrix where the main diagonal represents the zero distance between each variable from itself.

**The Contractive Factor**

Another way to interpret the squared weights matrix of the AutoCM system is to assume each variable of the dataset as a vector composed of all its values. Then, the dynamic value of each connection between two variables represents the local velocity of their mutual attraction caused by their mutual vectors similarity, with increasing similarity, increasing the attraction speed between the vectors. When two variables are attracted by each other, they contract proportionally to the original Euclidean space between them. The limit case is when two variables are identical, in this case the space contraction should be infinite and the two variables should collapse at the same point.

We can extract from each weight of a trained AutoCM this specific contractive factor:

(9a) $\begin{matrix} F_{i,j}=\left(1-\frac{w_{i,j}}{C}\right)^{-1}; \\ 1\leq F_{i,j}\leq\infty. \end{matrix}$

This is interesting for the following reasons:

1. it is the inverse of the equation used as the contractive factor during the AutoCm training;
2. considering equation (3b) , each mono-connection $v_{i}$ at the end of training will reach the value C. In this case the contractive factor will be infinite because the two variables connected by the weight are the same variable.
3. considering equation (7b), each weight $w_{i,j}$, at the end of training will always be smaller than C. This means that the contractive factor for each weight of the matrix that we consider will always be non-infinite. In fact, in the case of the weight$w_{i,i}$, when the variable is connected with itself, the variable has also received the influences of all other variables (the matrix $\mathbf{w}$is a squared matrix where each variable is linked to the other). Consequently, this variable has not remained exactly the same.

At this point, we can calculate the contractive distance between each variable and any other, modifying the original Euclidean distance with a specific contractive factor.

The Euclidean distance among the variables in the dataset is given by the following equation:

(10a) $\begin{matrix} d_{i,j}^{[Eucliden]}=\sqrt{\sum_{k}^{R} (x_{i,k}-x_{j,k})^{2}}; \\ \text{where :} \\ R=\text{ the number of the records of the assigned dataset;} \\ x_{i,k}\text{ and }x_{j,k}=\text{ the i-th value and the j-th value of two variables in the k-th record.} \end{matrix}$

And, consequently, the AutoCM distance matrix among the same variables is:

(11a) $d_{i,j}^{[AutoCM]}=\frac{d_{i,j}^{[Euclidean]}}{F_{i,j}}.$

**A conceptual description of minimum spanning tree concept**

A minimum spanning tree (MST) is a spanning tree of a connected, undirected graph. It connects all the vertices together with the minimal total weighting for its edges.

A single graph can have many different spanning trees. We can also assign a weight to each edge, which is a number representing how unfavorable it is, and use this to assign a weight to a spanning tree by computing the sum of the weights of the edges in that spanning tree. A minimum spanning tree or minimum weight spanning tree is then a spanning tree with weight less than or equal to the weight of every other spanning tree.

There are quite a few use cases for minimum spanning trees. One example would be a telecommunications company which is trying to lay out cables in new neighborhood. If it is constrained to bury the cable only along certain paths (e.g. along roads), then there would be a graph representing which points are connected by those paths. Some of those paths might be more expensive, because they are longer, or require the cable to be buried deeper; these paths would be represented by edges with larger weights. Currency is an acceptable unit for edge weight – there is no requirement for edge lengths to obey normal rules of geometry such as the triangle inequality. A spanning tree for that graph would be a subset of those paths that has no cycles but still connects to every house; there might be several spanning trees possible. A minimum spanning tree would be one with the lowest total cost, thus would represent the least expensive path for laying the cable.

In bio-medical field the MST has been used particularly in microarray clustering. Although MST-based clustering is formally equivalent to the dendrograms produced by hierarchical clustering under certain conditions; visually they can be extremely different.


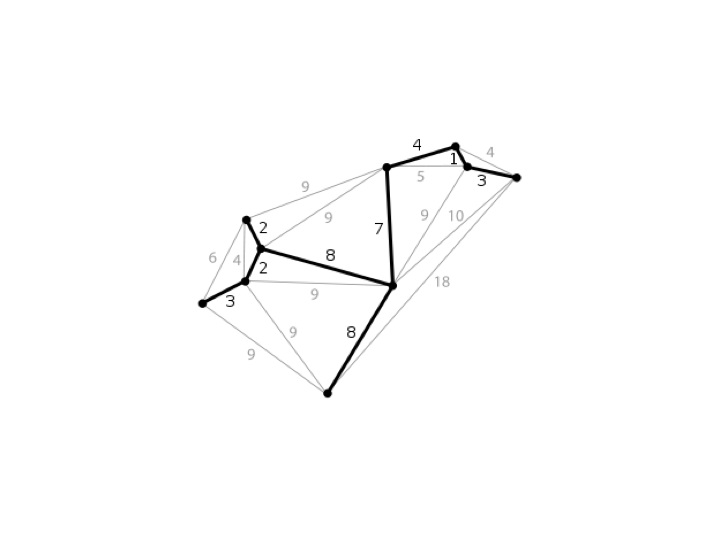


**
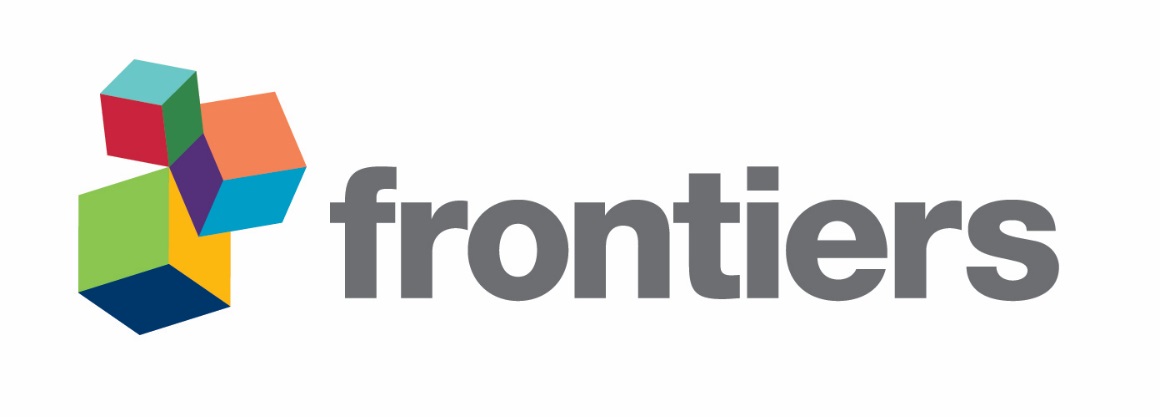
**
